# An accurate and interpretable model for siRNA efficacy prediction

**DOI:** 10.1186/1471-2105-7-520

**Published:** 2006-11-30

**Authors:** Jean-Philippe Vert, Nicolas Foveau, Christian Lajaunie, Yves Vandenbrouck

**Affiliations:** 1 Centre for Computational Biology, Ecole des Mines de Paris, 35 rue Saint-Honoré, 77300 Fontainebleau, France; 2 Laboratoire de Biologie, Informatique, Mathématiques, Département Réponse et Dynamique Cellulaire, CEA Grenoble, 17 rue des Martyrs, 38054 Grenoble, France

## Abstract

**Background:**

The use of exogenous small interfering RNAs (siRNAs) for gene silencing has quickly become a widespread molecular tool providing a powerful means for gene functional study and new drug target identification. Although considerable progress has been made recently in understanding how the RNAi pathway mediates gene silencing, the design of potent siRNAs remains challenging.

**Results:**

We propose a simple linear model combining basic features of siRNA sequences for siRNA efficacy prediction. Trained and tested on a large dataset of siRNA sequences made recently available, it performs as well as more complex state-of-the-art models in terms of potency prediction accuracy, with the advantage of being directly interpretable. The analysis of this linear model allows us to detect and quantify the effect of nucleotide preferences at particular positions, including previously known and new observations. We also detect and quantify a strong propensity of potent siRNAs to contain short asymmetric motifs in their sequence, and show that, surprisingly, these motifs alone contain at least as much relevant information for potency prediction as the nucleotide preferences for particular positions.

**Conclusion:**

The model proposed for prediction of siRNA potency is as accurate as a state-of-the-art nonlinear model and is easily interpretable in terms of biological features. It is freely available on the web at

## Background

RNA interference (RNAi) is the process through which a double-stranded RNA (dsRNA) induces gene expression silencing, either by degradation of sequence-specific complementary messenger RNA (mRNA) or by repression of translation [1]. The RNAi pathway was firstly identified in lower organisms (plants, fungi and invertebrates) and led to many successful applications such as genome-wide RNAi screens [2-5]. In mammalian systems, chemically synthesized dsRNA reagents shorter than 30 nt were found to trigger sequence-specific RNAi response without inducing the cell's immune mechanism [6,7]. The use of exogenous small interfering RNAs (siRNAs) for abolishing gene expression has quickly become a widespread molecular tool providing a powerful means for gene functional study and new drug target identification [8,9]. Moreover, RNAi represents a promising technology for therapeutic applications against HIV [10], neurodegenerative disorders [11] and cancer [12]. Its popularity stems in particular from its simplicity and low cost compared to other methods, e.g., involving knockout mice.

Considerable progress has been made recently in understanding how the RNAi pathway mediates gene silencing. Two main types of sequence-specific cleavage triggers have been identified: siRNAs and micro RNAs (miRNAs), chemically synthesized respectively from long dsRNA and miRNA precursor (pre-miRNA) by Dicer, a multidomain enzyme of the RNase III family. Once synthesized the siRNA/miRNA is incorporated into a ribonucleoprotein complex (RNP) called RISC loading complex (RLC). Duplex is unwounded and one strand is selectively incorporated into the RNA-induced silencing complex (RISC). Then this complex triggers either mRNA degradation or translational repression of the mRNA depending on the degree of complementarity between the RISC-associated RNA strand (the guide strand) and the target. Although miRNAs differ from siRNAs in their biogenesis, their functions are highly similar if not identical [13].

siRNA design is one of the most crucial steps in reliable use of RNAi, since it must ensure the efficacy and the specificity of the selected sequence for a target gene [9,14]. Tuschl et al. [15] provided a set of guidelines (commonly known as the MPI principles) on how to design effective siRNA. These empirical rules, based for example on GC content and symmetric 3' TT overhangs, are however not discriminative enough since significant proportion of ineffective siRNAs following these rules were reported [16]. Recent advances in the understanding of the biochemical mechanism of RNAi and statistical analyses of experimentally verified siRNAs have highlighted new biochemical and biophysical features of the siRNA reagents. It has been shown that thermodynamic profiles of the siRNA duplex determine which strand enters RISC as the guide strand and that the antisense strand can only direct cleavage of the sense mRNA targets [17,18]. These functionally asymmetric siRNA duplexes exhibit lower base-pairing stabilities at the 5' end of the antisense strand and at the cleavage site. It was also suggested by either experimental or computational means that mRNA target accessibility could contribute to silencing activity [19-21], although the extent of this contribution remains controversial [22,23]. Moreover, off-targets effects of siRNAs to unrelated mRNA targets were observed in several studies, which partially explain the loss of potency in silencing effect [24,25]. Several studies combining cellular assays and statistical analysis reported features that consistently correlate with functionality across data sets of experimentally validated siRNAs. For instance, Ui-Tei et al. [26] proposed several criteria that correlate with highly effective silencing after an analysis of 62 siRNAs targeting six genes, including for example the presence of A/U at the 5' end and of G/C at the 3' end of the antisense strand. Amarzguioui and Prydz [27], analyzing a dataset of 80 siRNAs duplexes targeting four genes, corroborated these findings and expanded them by identifying sequence motifs on the siRNA sense strand that correlated positively (S1, A6, W19) and negatively (U1, G19) with potency at 70% knockdown level. Finally, on the basis of a systematic analysis of 180 siRNAs that target two mRNA regions, Reynolds et al. [28] proposed eight parameters that taken together increase the probability of selecting an effective siRNA and further incorporated them into a widely-used rational design algorithm.

More recently several programs and websites have been developed for automatic siRNA design, implementing design rules based on sequence features, hairpin potential formation, stability profiles, energy features, weighted patterns and mRNA secondary structure [23,29]. A comparison study of these methods on a larger collection of siRNAs has however shown that many of them give close to random classification on unseen data [30]. According to the current best rational design approach, less than 35% of siRNAs experimentally tested produce more than 90% of gene silencing, and almost 20% of siRNAs result in less than 50% efficacy [31]. Therefore, despite considerable efforts, the design of high efficacy siRNA is far from conclusive. The variability in performance of potency prediction algorithms could be explained by the limited amount of siRNA sequences used during their design, together with the fact that these limited datasets are often biased since many published siRNAs have been designed following the MPI or Reynolds' rules [32].

In this context the publication in Huesken et al. [33] of an unbiased set of 2431 randomly selected siRNAs targeting 34 mRNA species assayed through a high-throughput fluorescent reporter gene system, together with the potency prediction system BIOPREDsi based on an artificial neural network trained on this dataset, represents a landmark. With a reported Pearson correlation coefficient of 0.66 between the measured and predicted efficacy on a set of 249 siRNAs not used during training, BIOPREDsi was shown to be more accurate than previous simpler models. The biological interpretation of the function encoded by BIOPREDsi is however challenging due to the use of nonlinear terms, and was not discussed in Huesken et al. [33].

In this study we investigate the possibility to design both an accurate and easily interpretable model to predict siRNA potency. To ensure direct interpretability of the model we restrict ourselves to linear models over two simple sets of features of siRNA sequences: the nucleotides present at each position in the siRNA sequence and the global content of the siRNA in short motifs. In order to allow a potentially large number of features and focus only on the most informative of these features, we train the linear model on the dataset of Huesken et al. [33] with the LASSO regression method [34] which leads to sparse linear models, automatically discarding irrelevant features if any. We show that both representations contain relevant and partially complementary information to predict the siRNA potency, and that the combination of both representations leads to a simple linear model as accurate as the BIOPREDsi neural network, with a Pearson correlation coefficient of 0.67 on the dataset used to evaluate the performance of BIOPREDsi. The analysis of the weights of the linear model allows a comprehensive description of the influence of various features in the siRNA sequence related to its efficacy. We show that only few features are in fact irrelevant, detect and quantify known and new preferences of nucleotides at several positions, and observe the importance of the presence of asymmetric short motifs in the siRNA sequence with A/U at the 5' end and C/G at the 3' end of the guide strand. We notice particularly that the inclusion of the 2 overhang nucleotides at the 3' end of the guide strand in the model improves prediction accuracy. We also show that models based on the thermodynamic features are significantly less accurate than those based on the sequence features only, highlighting the limitations of these features.

## Results

### Representation of siRNA sequences

In order to develop an interpretable linear model for siRNA efficacy prediction we must chose a set of features to represent siRNA sequences. By convention we will always refer to the nucleotides in the antisense guide strand below, with positions in the sequence ordered in the 5'→3' direction on the guide strand. The siRNA sequences can therefore formally be represented as strings of length 21 in this study (including two 3' overhang nucleotides at positions 20 and 21). Previous studies have highlighted the importance of at least two families of string features that largely determine siRNA potency. First, several authors have observed that preferred nucleotides at specific positions are strong indicators of siRNA efficacy: for example, A and U are overrepresented in the first position of the guide strand of potent siRNAs, while C is overrepresented in positions 7 and 11 of siRNAs with poor efficacy [18,28,33]. We therefore define the *sparse-21 *representation of a siRNA sequence as the binary vector of dimension 84 that indicates the presence (1) or absence (0) of each nucleotide at each of the 21 positions. The influence of the two 3' overhang nucleotides being controversial, we also consider the sparse representation restricted to the first 19 nucleotides, called *sparse-19 *below. It is a binary vector of dimension 76 that encodes the presence or absence of each nucleotide at each of the first 19 positions. Second, Teramoto et al. [35] has shown that the composition of siRNAs in short motifs of length 1 to 3, without positional information, also contains relevant information for efficacy prediction. We therefore define the *spectral *representation of a siRNA sequence as the vector of count of occurrences of each nucleotide motif of length 1 to 3. There are respectively 4, 16 and 64 possible nucleotide motifs of length 1, 2 and 3, so the spectral representation of a siRNA guide sequence is an integer-valued vector of dimension 4 + 16 + 64 = 84. As for the sparse representation, we call *spectrum-19 *and *spectrum-21 *the spectral representation for the 19- and 21-length siRNA sequence, respectively. Finally, we define the *composite *representation of a siRNA sequence as the concatenation of the sparse and spectral representations. The composite representation is a vector of dimension 160 or 168, depending on whether it is based on the sparse-19 or sparse-21 representation. It provides the simplest way to integrate the potentially complementary information provided by the presence of specific nucleotides at specific positions, on the one hand, and the global content of particular short motifs, on the other hand.

### Accuracy of siRNA potency prediction

We estimated a linear model based on the various sparse, spectral and composite representations from the siRNA datasets provided by Huesken et al. [33]. This dataset comprises a total of 2431 randomly selected siRNAs targeting 34 mRNAs, split into a training set of 2182 sequences used to train a predictive model, and a test set of 249 sequences to evaluate the performance of the predictive model. Given the large dimensions of the vectors compared to the limited number of sequences available for training, we estimated the linear model with the LASSO procedure which is known to be resistant to large dimensions [34]. If only a few variables are relevant the LASSO has also the good property of shrinking the weights of irrelevant features to zero, therefore providing simpler interpretation of relevant features in the final model. Table 1 shows the performance of the predictive linear models trained on the various sparse, spectral and composite representations. The performance is assessed in terms of Pearson correlation coefficient between the predicted and the true efficacy for the 249 siRNAs in the test set, in order to provide a fair comparison with Huesken et al. [33] where the neural-network-based BIOPREDsi predictive engine (based on the sparse-21 representation) has a reported performance of 0.66.

**Table 1:** Performance of different representations of siRNA sequences

Representation	Correlation
Sparse-19	0.62
Spectrum-19	0.64
Composite-19/19	0.64
Sparse-21	0.65
Spectrum-21	0.58
Composite-21/21	**0.67**
Composite-21/19	**0.67**
Free energy profile	0.54
Stability profile	0.53
BIOPREDsi [33]	0.66

This table shows the Pearson correlation coefficients between the predicted and true efficacy on the test set for linear models estimated on the training set with the LASSO procedure and different representations of siRNA sequences. The representation Composite-xx/yy corresponds to the combination of the Sparse-xx and Spectral-yy representations. Additionally the performance of the BIOPREDsi neural network [33] is included for comparison.

**Figure 1:**
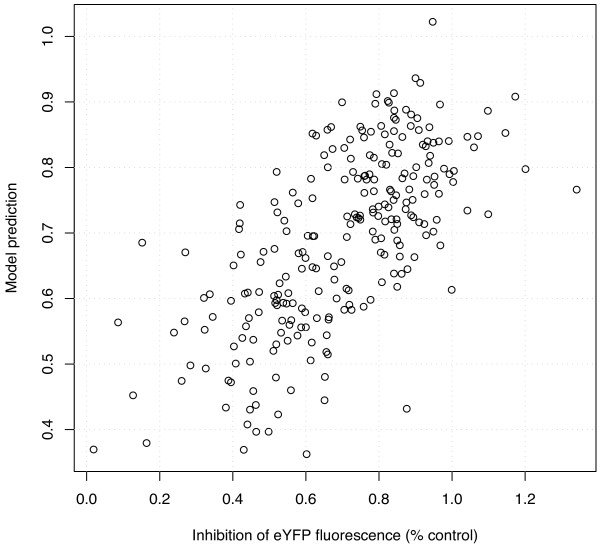
**Accuracy of the model**. This plot shows the predicted versus actual silencing efficacy of siRNA in the test set of 249 siRNA, for a LASSO model trained on the composite-21 representation of the 2182 training data set.

As observed in Huesken et al. [33], the inclusion of the two 3' overhang nucleotides of the guide strand in the sparse representation leads to more accurate models (0.65 vs. 0.62 for the sparse-21 vs. sparse-19 representations), suggesting that complementariness over the full oligoribonucleotide guide length provides an improved gene knock-down. Although less intuitive, the spectral-19 representation outperforms the sparse-19 representation (0.64 vs. 0.62), confirming the good results claimed by Teramoto et al. [35] on smaller datasets. This shows that, although much previous work has focused on position-dependent features of siRNA (in particular nucleotide preferences and local thermodynamic features), position-independent short motif contents can provide at least as much predictive power. Surprisingly, the spectral representation of the 21-length siRNA (including antisense 3' overhangs) is clearly worse than the spectral representation of the 19-length siRNA (without antisense 3' overhang). This suggests that mixing the motif content of the first 19 nucleotides with that of the antisense 3' overhang tends to blur the information contained in the spectral representation for efficacy prediction, a point to be clarified below when we examine the weights associated to the different features. Interestingly, the composite representations integrating the 19 or 21 sparse and spectral representations always perform at least as well as (when antisense 3' overhangs are discarded) or better than (when antisense 3' overhangs are included) the best among the spectral and sparse representations alone. This confirms that the sparse and spectral representations contain slightly complementary relevant information, and that the LASSO procedure is able to extract this information from the composite representation in spite of the large dimension of the vectors. The discrepancy between the actual and predicted efficacy for the 249 siRNAs in the test set for the linear model trained on the composite-21/19 representation (i.e., concatenation of the sparse-21 with the spectral-19 representations) is further illustrated in Figure 1.

In terms of absolute precision, we observe that the performance of the simple linear models is competitive with the nonlinear BIOPREDsi prediction method. Based on the similar sparse-21 representation, the linear model has a correlation coefficient of 0.65, to be compared with the 0.66 correlation reported by Huesken et al. [33]. This suggests that the gain resulting from the possible nonlinearity of the neural network is at most marginal in this application, and that the relationship between the siRNA features we and other have considered and the experimental efficacy of the siRNA can accurately be described by an affine model. Furthermore we observe that the composite representations that add the spectral (19 or 21) to the sparse-21 representation result in slightly better performance than the BIOPREDsi model (0.67 vs. 0.66), therefore confirming that this simple approach is state-of-the-art in terms of performance on this dataset. In order to further assess the performance of the linear model based on the composite-21/19 representation, we repeated three times a full 10-fold cross-validation experiment on the 2431 siRNA. The average correlation coefficients over the 30 train/test splits was 0.657, with a standard deviation of 0.039, confirming that the particular train/test split defined by Huesken et al. [33] provides a fair estimate of the performance of the methods. We also trained and tested the LASSO approach on the various training and test sets described by Huesken et al. [33]. Table 2 shows the results of this experiments, together with the performance of BIOPREDsi in the same context, and confirms the good performance of the linear model.

**Table 2:** Comparison of the LASSO model and the BIOPREDsi algorithm on difference training and test sets.

Training Set	All (249)	All human (198)	hE2 (139)	Rodent (51)
All (2,182)	**0.67**/0.66	**0.66**/0.63	**0.67**/0.63	0.75/**0.77**
All human (1,744)	**0.66**/0.65	**0.65**/0.61	**0.66**/0.62	**0.72**/**0.72**
Human E2s (1,229)	**0.65**/**0.65**	**0.64**/0.62	**0.65**/0.62	0.71/**0.76**
Rodent (438)	**0.57**/0.55	**0.55**/0.54	**0.55**/0.53	**0.68**/0.57
Random all (1,091)	**0.66**/0.65	**0.64**/0.62	**0.64**/0.61	**0.76**/0.75
Random all (727)	**0.67**/0.65	**0.66**/0.63	**0.66**/0.63	0.74/**0.76**
Random all (545)	0.61/**0.62**	**0.61**/0.60	**0.61**/0.60	0.65/**0.70**
Random all (218)	**0.57**/0.47	**0.56**/0.47	**0.55**/0.46	**0.60**/0.46

This table shows the Pearson correlation coefficients between predicted and actual efficacy on training and test data sets of various sizes and composition. Each cell reports two values. The first one is the coefficient obtained by the LASSO method with the combination of the sparse representation on 21 nucleotides and the spectral representation on the first 19 nucleotides only. The second value is the coefficient reached by BIOPREDsi on the same dataset (from Huesken et al. [33].)

On most tasks, in particular when the test set is made of human sequences ("All human") or of the human E2 genes ("hE2"), the linear model outperforms the neural network model by a few percents. The picture is less clear on test set made of rodent sequences ("Rodent"), where the linear and nonlinear models are closer to each other. Interestingly, with very few training examples (218 sequences), the performance of the LASSO linear model with the composite-21/19 model clearly outperforms the nonlinear neural network (by 0.09 to 0.14 on the four test sets) in spite of the large dimension of the vectors (168), confirming the relevance of the regularized linear LASSO procedure in this context.

### Interpretability of the prediction

**Figure 2:**
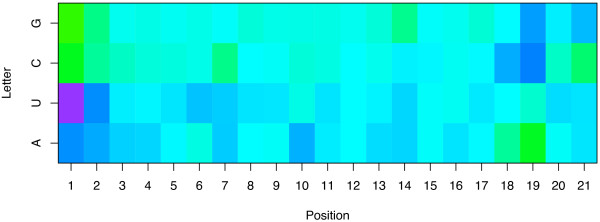
**Weights of the model trained on the sparse-21 representation**. The picture shows the weights of the linear model estimated by the LASSO on the sparse-21 representation. Each column corresponds to one position in the siRNA, numbered from 1 to 21 in the 5'→3' order the antisense strand, each row to a nucleotide of the guide strand. The color of a cell represents the weight associated with a given nucleotide at a given position: blue colors indicate a tendency to increase the efficacy of the siRNA, green colors a tendency to decrease it.

The model built with the LASSO procedure being linear, it is possible to understand how the predicted accuracy is computed and to assess the importance of the different features by examining the weights of the model associated to each feature. For example, for the sparse representation a weight is attributed to each nucleotide at each position, and the efficacy predicted for a siRNA is simply the sum of the weights of the nucleotides of the siRNA. Table 3 presents the weights attributed to the features of the sparse-21 representation when the LASSO is trained on the training set of 2182 siRNA sequences, and Figure 2 displays them graphically. We recognize in this picture several well-known features mentioned in previous studies, as well as new ones. The two main differences between previously reported nucleotide features and the features highlighted in Table 3 and Figure 2 are that (i) the features obtained by the LASSO result from a global analysis of the complete dataset, as opposed to statistical analysis of subsets of siRNAs with extreme potencies (e.g., the top and bottom 8% siRNAs in terms of accuracy in the case of Huesken et al. [33]), and (ii) we provide a precise quantitative assessment of the importance of each feature, the weight of a feature being its contribution in the final predicted silencing efficacy. For example, the respective weights of A, U, C and G in the first guide strand position are 10.94, 18.39, 0 and -2.30, showing that the most preferred nucleotide in the first position is U, and that replacing a G by a U has an expected effect of increasing the efficacy by 21% (when the efficacy is measured in percentage of inhibition of eYFP fluorescence compared to a control experiment, as described in Huesken et al. [33]). The weights reflect the required asymmetry already detected and discussed [17,18,33], with a strong propensity for active siRNAs to contain a A or a U at the 5'-guide terminus (positions 1 and 2), a C or a G at position 19, and a C at position 18. Using a different methodology to compare the 8% most active with the 8% least potent siRNAs, Huesken et al. [33] detected the overrepresentation of U at position 2, but not that of A at the same position, concluding that this feature does not reflect the need for a weak bond close to the 5'-end.  Our findings based on the analysis of all data simultaneously however detect the importance of U as well as A, suggesting that this feature can simply be related to the need for weak affinity at this position. More generally, a global propensity to contain more A and U toward the 5'-end and more C and G towards the 3'-end of the guide strand emerges from this picture. On top of this general trend, several particular positions have large weights for particular nucleotides. As already observed by Huesken et al. [33], the requirement for an A at position 10 corresponds to a previously characterized U-cleavage site [36].

**Table 3:** Weights of the linear model trained on the sparse-21 representation.

Feature	Weight	Feature	Weight	Feature	Weight	Feature	Weight
A1	10.94	C1	0	G1	-2.30	U1	18.39
A2	0	C2	-6.67	G2	-7.28	U2	0.97
A3	1.10	C3	-2.74	G3	-1.23	U3	0
A4	1.19	C4	-1.67	G4	-1.25	U4	0
A5	0.60	C5	-0.78	G5	0	U5	1.32
A6	-0.25	C6	0	G6	0	U6	3.10
A7	0.11	C7	-5.86	G7	-2.02	U7	0
A8	0	C8	0	G8	-1.42	U8	0.95
A9	0	C9	0	G9	-0.55	U9	1.17
A10	3.85	C10	-0.50	G10	0	U10	0
A11	1.69	C11	-0.08	G11	0	U11	1.99
A12	0	C12	0	G12	-0.59	U12	0.01
A13	0.88	C13	-1.06	G13	-1.81	U13	0
A14	1.09	C14	0	G14	-4.37	U14	1.04
A15	0	C15	0.48	G15	0	U15	0
A16	1.35	C16	0	G16	-0.24	U16	0
A17	0	C17	0	G17	-1.70	U17	0.62
A18	-3.61	C18	2.99	G18	0	U18	-0.04
A19	-5.04	C19	6.31	G19	5.32	U19	0
A20	-0.91	C20	-2.64	G20	0	U20	0.65
A21	-0.05	C21	-5.83	G21	1.56	U21	0

This table gives the weights of the linear model trained on the sparse-21 representation to predict the siRNA efficacy (offset = 66.9).

**Figure 3:**
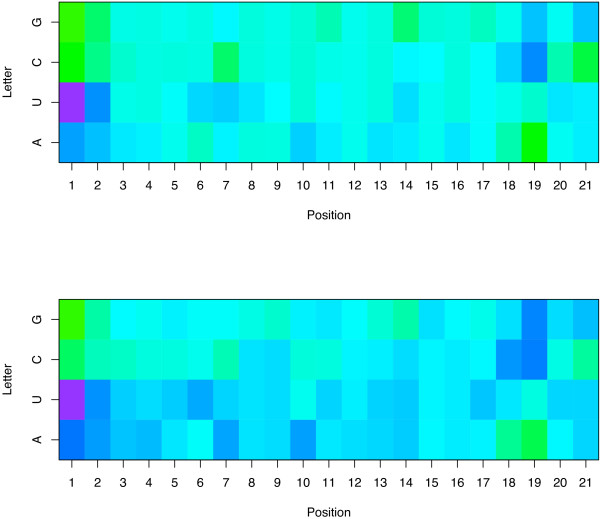
**Weights of two models trained on the sparse-21 representation from two independent training sets**. The picture shows the weights of two linear models trained on two independent sets of siRNA, with the same numbering and coloring conventions as Figure 2. We observe the conservation of many motifs between the two models.

Conversely, several nucleotides tend to have a strong negative effect on the potency of siRNA, including C at positions 7 and 21, and G at position 14. The pattern observed at positions 20 and 21 suggests a strong negative effect of C at these positions, and explains why more accurate models are obtained from the sparse representation of 21 nucleotides than from that of 19 nucleotides. Interestingly there is a big difference between the weights of C and G in the antisense 3' overhang positions, confirming that this feature is not merely related to the need for weak or strong hybridization in this position (as in the 5' end) but rather to individual properties of nucleotides. Finally, more subtle motifs appear in this picture and might suggest biological interpretations, such as the positive effect of A in position 3, 4 and 16, and of U in positions 5 to 9, 11, 14 and 17. In order to check the robustness of these motifs with respect to the data used to train the algorithm, we randomly split the training set into two non-overlapping subsets, and trained a model with the LASSO on both subsets independently. The resulting weights are graphically shown in Figure 3. The comparison of the two models learned to each other highlights the conservation of most motifs discussed above, suggesting that they are not just an artifact of the training set but might be related to some biological function.

**Figure 4:**
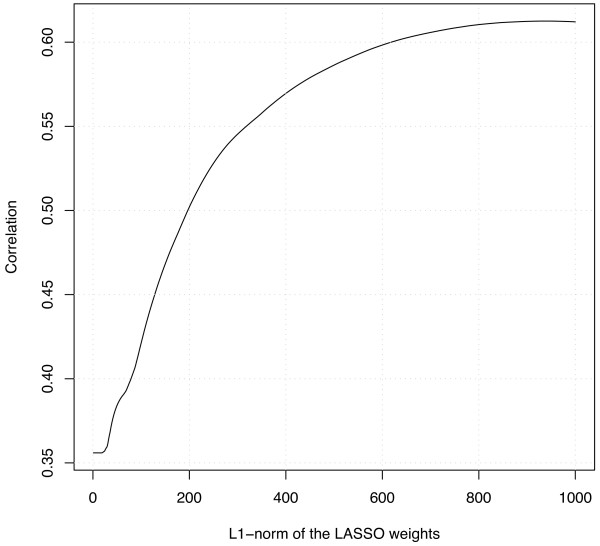
**Importance of the number of features**. This curve shows the accuracy obtained by the LASSO in a cross-validation as a function of the L1 norm of the weight vector. Small L1 norms generally correspond to sparse models involving a small number of features. The fact that this curve reaches its maximum for large L1 norms shows that most if not all features contribute to the accuracy of the model.

In fact, as observed in Table 3, very few positions seem to be without influence on the efficacy of the siRNA. A question worth investigating is whether all the features appearing in Table 3 and Figure 2 really help predict efficacy, or whether some of them may be discarded. The fact that the LASSO model tries to find parsimonious models based on as few features as possible to predict accuracy suggests that all detected motifs indeed play a role. As an illustration, Figure 4 shows the accuracy obtained by the LASSO in a cross-validation experiment as a function of the L1 norm of the weight vector, which is closely related to the number of non-zero weights. The fact that the performance of the LASSO increases almost until the weights reach the ordinary least square solution shows that increasing the number of features used in prediction indeed improves the accuracy of the prediction. Of course, only the most evident predictive features are detected and used when few siRNA sequences are available for training, but the conclusion so far is that if enough siRNA sequences are available for training, then most if not all features are likely to contribute either positively or negatively to the efficacy of siRNA. This suggests in particular that as more siRNA data become available, the most precise efficacy prediction models for siRNA design will have to rely on most if not all features simultaneously. We note that this conclusion is only valid in the framework of the method we use for feature selection (constraint of the L1 norm of the weight vector), and that other feature selection procedures might lead to different conclusion; only biological validation could confirm the hypothesis that most positions influence the efficacy of siRNA.

A similar analysis can be performed on the features of the spectral representation. In brief, Table 4 summarizes the weights associated to the spectral features when the LASSO is trained on the spectral features of the first 19 nucleotides. This table shows that, more than a general enrichment in particular nucleotides, the LASSO on the spectral representation detects the asymmetry of the guide sequences. Indeed, the main positive weights involve motifs with A or U at their 5' end, C or G at their 3' end (e.g., AAC, UC, UG, AAG, AGC), while the main negative weights also involve asymmetric motifs but with opposite direction (e.g., CUU, CUA, GUU, GU, GAU). For example, the motif UG has the third-largest positive weight, while GU has the fifth most negative weight. A notable exception to this strong trend are the motifs CUC and CUG that have strong negative weights in spite of no obvious asymmetry. The comparison of these weights with the motifs on Figure 2 highlights the reason why, contrary to the sparse representation, the spectral representation should be restricted to positions 1 to 19 of the antisense sequence. Indeed, positions 20 and 21 contain relevant information used by the sparse representation.

**Table 4:** Weights of the linear model trained on the spectral-19 representation.

Feature	Weight	Feature	Weight	Feature	Weight	Feature	Weight
A	0	C	0	G	-1.77	U	5.12
AA	0	AC	7.32	AG	7.11	AU	0
CA	-3.50	CC	0	CG	0	CU	-1.51
GA	-4.46	GC	0	GG	0	GU	-7.44
UA	0.35	UC	10.21	UG	10.12	UU	0
AAA	0.25	AAC	11.41	AAG	8.90	AAU	1.14
ACA	0.25	ACC	4.61	ACG	2.59	ACU	-0.53
AGA	0	AGC	7.64	AGG	5.46	AGU	1.83
AUA	0	AUC	3.12	AUG	1.68	AUU	-3.19
CAA	-4.93	CAC	1.81	CAG	0	CAU	-5.77
CCA	-5.62	CCC	-1.87	CCG	1.88	CCU	-5.69
CGA	-6.02	CGC	1.61	CGG	0	CGU	-3.21
CUA	-9.14	CUC	-9.09	CUG	-7.04	CUU	-13.06
GAA	-5.03	GAC	2.67	GAG	1.67	GAU	-6.17
GCA	-4.31	GCC	0	GCG	0.63	GCU	-4.24
GGA	-2.27	GGC	1.23	GGG	0.69	GGU	-1.51
GUA	-4.20	GUC	-1.64	GUG	-2.00	GUU	-8.85
UAA	-0.55	UAC	5.03	UAG	7.17	UAU	0
UCA	-3.34	UCC	3.68	UCG	7.53	UCU	-0.82
UGA	0	UGC	3.70	UGG	2.87	UGU	0
UUA	-0.39	UUC	1.97	UUG	4.34	UUU	-3.85

This table gives the weights of the linear model trained on the spectral-19 representation (offset = 39.3).

However they break the general trend of having a decreasing frequency of A-U and increasing frequency of C-G towards the 3'-end, with for example a C at position 21 appearing as a strong handicap for siRNA potency. This in turns implies that the motifs detecting the asymmetry of the sequence are less likely to be good predictors. For example, the motif AAC is the strongest positive predictor of potency when restricted to the first 19 nucleotides, but the presence of AAC at positions 18–20 or 19–21 is a strong inhibitor of siRNA potency. The fact that the spectral-19 representation even outperforms the sparse-19 representation suggests that the presence of asymmetric motifs along the siRNA sequence is at least as important as the presence of particular nucleotides at given positions for efficacy prediction. Because these two types of features contain independent information (nucleotide preferences at particular positions and presence of short asymmetric motifs with A/Us followed by C/Gs in the 5' to 3' guide strand direction), it is no surprise that the composite representation, that integrates both types of features, performs at least as well as each type of features taken independently. For sake of completeness the weights of the model trained on the composite-19/21 representation are presented in Table 5.

**Table 5:** Weights of the linear model trained on the composite-19/21 representation.

Feature	Weight	Feature	Weight	Feature	Weight	Feature	Weight
A	0	C	0	G	0	U	0
AA	0	AC	0.03	AG	0	AU	0
CA	0	CC	-0.38	CG	-0.26	CU	0
GA	0	GC	0	GG	-1.10	GU	0
UA	0.61	UC	0.33	UG	0	UU	0
AAA	-0.75	AAC	1.76	AAG	0.22	AAU	0
ACA	1.64	ACC	0	ACG	-1.09	ACU	1.08
AGA	-0.30	AGC	0.21	AGG	0	AGU	0.10
AUA	0	AUC	0.61	AUG	-0.24	AUU	0
CAA	0	CAC	0	CAG	0	CAU	0
CCA	0	CCC	-2.81	CCG	0	CCU	0
CGA	-0.63	CGC	0	CGG	-0.11	CGU	0
CUA	0	CUC	-1.12	CUG	0	CUU	0
GAA	0	GAC	0	GAG	0	GAU	-0.69
GCA	0	GCC	-2.26	GCG	-1.67	GCU	0
GGA	0	GGC	-2.08	GGG	-0.97	GGU	0
GUA	0	GUC	0.20	GUG	0	GUU	-1.20
UAA	0	UAC	0	UAG	1.71	UAU	0.13
UCA	0	UCC	0	UCG	2.51	UCU	1.78
UGA	0.92	UGC	0	UGG	0	UGU	0.53
UUA	0	UUC	0	UUG	1.72	UUU	-0.10

A1	10.40	C1	0	G1	-1.90	U1	17.23
A2	0	C2	-5.72	G2	-5.56	U2	0.71
A3	0.76	C3	-1.72	G3	0	U3	0
A4	1.40	C4	-0.35	G4	0	U4	0
A5	0	C5	-0.31	G5	0	U5	0
A6	-1.78	C6	0	G6	0	U6	0.91
A7	0.22	C7	-4.16	G7	-0.28	U7	0
A8	-0.45	C8	0.04	G8	-0.23	U8	0
A9	-1.03	C9	0.51	G9	-0.06	U9	0
A10	2.81	C10	0	G10	0.96	U10	-0.96
A11	0	C11	0	G11	0	U11	0.01
A12	0	C12	0.20	G12	0	U12	0
A13	0.92	C13	0	G13	-0.31	U13	0
A14	0.05	C14	0	G14	-3.76	U14	0
A15	-1.48	C15	0.50	G15	0	U15	-1.01
A16	0.27	C16	0	G16	0	U16	-1.36
A17	0	C17	0.74	G17	-0.08	U17	0
A18	-4.92	C18	2.92	G18	0	U18	-1.43
A19	-5.39	C19	7.32	G19	5.26	U19	0
A20	-1.16	C20	-2.77	G20	0	U20	0.49
A21	0	C21	-5.90	G21	1.46	U21	0

This table gives the weights of the linear model trained on the composite-19/21 representation of the siRNA (offset = 69.3).

### Thermodynamic features

Previous studies have insisted on the correlation between the stability asymmetry of the siRNA sequence and the efficacy of gene silencing. It has been observed that thermodynamic stability profile of the siRNA duplex determines which strand enters the RISC complex as guide strand, and that the most potent siRNA duplexes therefore present lower base-pairing stabilities at the 5' end of the guide strand, as well as at the cleavage site [17,18]. This suggests that local stability profiles and base-stacking energy profiles might be relevant features to help predict the efficacy of siRNA. Table 1 shows the result of siRNA efficacy prediction when these features are used. In both cases the correlation obtained is in the range 0.54, confirming that these features are indeed simple and good predictors. However we note that they provide less precise models than when larger sets of features are used (e.g., the composite representation). We also observed that the combination of these profiles with the composite representations does not improve over the composite representation alone, suggesting that the asymmetry is already summarized in the sparse and spectral representations. Intuitively this latter point is not surprising given the nature of the spectral representation, which can be interpreted as the counts of hydrogen bounds and of the types of the nearest and the next-nearest stacking interactions in the siRNA oligomer, and of the sparse representation which gives information about the localization of the different nucleotides in the oligomer. Common models for the global Gibbs free energy of the oligomer are for example particular linear functions in the spectral representation. By allowing the LASSO to estimate *any *linear model, we give it the freedom to estimate a function that could be related to thermodynamic properties of the siRNA, although it could also be related to other properties. The performance we obtain suggest that a large part of the correlation captured by the LASSO model can in fact be related to thermodynamic properties, but that other features also contribute to siRNA efficacy. We note that recently Shabalina et al. [37] proposed a simple model for siRNA potency based only on a few compositional and thermodynamic features; its performance on the test set of Huesken et al. [33] reaches 0.52, slightly below the performance of our model based on thermodynamic features only (A. Ogurtsov, private communication).

**Figure 5:**
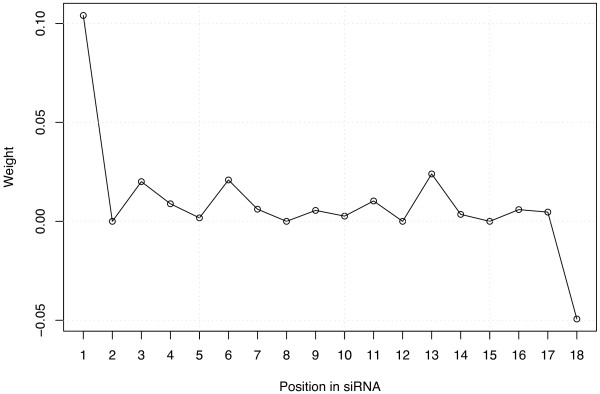
**Free energy profile**. This picture shows the weights of the linear models built by the LASSO from the free energy profile representation. It highlights the importance of the 3'- and 5'- stabilities for siRNA potency.

The weights of the linear model trained on the profile of pair stacking energies are shown in Figure 5. The local energies are particularly relevant at two positions of the siRNA for efficacy prediction : the first pair of nucleotide at the 5' end of the guide strand must have high stack energy, that is, be thermodynamically unstable for the siRNA to be potent, with an estimated average contribution of 10% gain in efficacy for each increase of 1 kcal/mol, while the last base pair at the 3' extremity must have a low free energy, that is be stable, with an estimated average contribution of -5% per increase of 1 kcal/mol in free energy. Smaller contributions of other features suggest that potent siRNAs should have a tendency for weak double-strand stability around positions 3–4, 6–7 and 13–14 of the guide strand.

### Performance on independent datasets

Several datasets published previous to and independently from the dataset of Huesken et al. [33] are available and provide a useful benchmark to assess the validity of our approach beyond the particularities of the dataset used to train the model. In this section we report results aiming at validating (i) the predictive accuracy of our model on independent datasets and (ii) the stability of the model interpretation with respect to the data used to train the model.

In order to assess the predictive accuracy of our model beyond Huesken's data we tested it on four independent datasets: the Reynolds dataset [28] of 240 siRNAs, the Vickers dataset [19] of 76 siRNAs, the Harborth dataset [38] of 44 siRNAs, and the compilation of 653 siRNA gathered by Shabalina et al. [37]. The first three datasets were used in [33] to assess the accuracy of BIOPREDsi on independent datasets, and therefore provide a useful comparison with BIOPREDsi. The fourth dataset of Shabalina et al. is a compilation of siRNA with experimentally measured activities gathered from 13 previous publications [37], and represents to our knowledge the most complete public compilation of siRNA data to date, apart from Huesken's data. In all cases only the first 19 nucleotides of the siRNA are available, so we tested the performance of the sparse-19, spectrum-19 and composite-19 models trained on the Huesken training set of 2182 siRNA. The results are presented in Table 6, and compared to the results of the BIOPREDsi algorithm in a similar setting for the first three datasets. Overall these results show no clear winner between the linear and neural network-based model, confirming that the models produced by the LASSO are robust to new datasets and competitive compared to BIOPREDsi. In terms of absolute values we observe a clear drop in the performance between the test set of 249 siRNA of Huesken (where the sparse-19, spectrum-19 and composite-19 have respective correlations of 0.62, 0.64 and 0.64) and the independent test sets (where the correlations range between 0.45 and 0.58). This drop in performance illustrates and quantifies the differences between Huesken's dataset and previous publications, both in terms of siRNA selection and in terms of efficacy measurements. We note in particular that Huesken et al. measure the knock-down efficacy by looking at protein levels [33], while in some other datasets the mRNA degradation is directly evaluated. The relatively high levels of correlation obtained nevertheless suggest that, beyond the many differences between various siRNA datasets, a coherent biological effect is quantified by the model.

**Table 6:** Performance on independent datasets.

Dataset	Sparse	Spectral	Composite	BIOPREDsi
Harborth (44)	0.43	**0.49**	0.48	0.45
Reynolds (240)	0.54	0.49	**0.55**	**0.55**
Vickers (76)	**0.58**	0.54	0.49	0.57
Shabalina (653)	**0.48**	0.45	**0.48**	-

This table shows the performance of the LASSO model with different features (sparse-19, spectrum-19, composite-19/19) on four independent datasets, as well as the performance of the BIOPREDsi algorithm on the the first three datasets (from Huesken et al. [33]). The performance is measured as the Pearson correlation coefficient between predicted and true efficacy).

**Figure 6:**
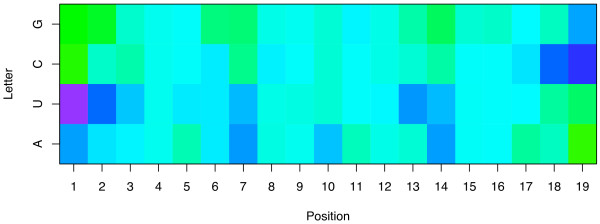
**Weights of the model trained on the sparse-19 representation of Shabalina et al.'s dataset**. The picture shows the weights of the linear model estimated by the LASSO on the sparse-19 representation trained on the 653 siRNA compiled by Shabalina et al. [37]. Each column corresponds to one position in the siRNA, numbered from 1 to 19 in the 5'→3' order the antisense strand, each row to a nucleotide of the guide strand. The color of a cell represents the weight associated with a given nucleotide at a given position: blue colors indicate a tendency to increase the efficacy of the siRNA, green colors a tendency to decrease it. A comparison of this picture with Figure 2 highlights the similarities and the differences of the LASSO model trained on independent datasets.

A second question of importance concerns the validity of the interpretation of the models trained on Huesken's dataset. In order to check whether the main features detected and analyzed in the previous sections are not merely a bias of this dataset, we trained the LASSO models based on the sparse-19, spectrum-19 and composite-19 representations on the Shabalina dataset of 653 siRNA. These models were tested on the Huesken test set of 249 siRNA, resulting in correlations of 0.60, 0.44 and 0.58, respectively. Compared to the correlations of 0.62, 0.64 and 0.64, respectively, obtained when the models are trained on the Huesken's training set of 2182 siRNA, we note a slight drop in performance for the sparse representation, and a more important one for the spectrum and composite ones. The important decrease in performance of the spectrum representation might be due in part to the limited number of sequences available for training (653 vs 2182), as we observed that the spectrum representation requires more examples than the spectral representation to be competitive. The good performance of the sparse representation (0.60 vs 0.62) suggests that the model trained on the Shabalina dataset has found biologically relevant features that are valid on Huesken's dataset. Figure 6 displays graphically the weights of the sparse-19 model trained on Shabalina's dataset. Compared to the weights of the spectrum-21 model trained on Huesken's training set (Figure 2), we observe numerous features shared between the two pictures, such as the importance of the features A/U in positions 1, 2 and 7 in potent siRNA. We also note a few differences, such as the presence of a U at position 13 which is a strong indicator of potency in Shabalina's data, but has a limited contribution to the model trained on Huesken's data. Many factors can explain the differences observed, such as the fact that many siRNA were designed following certain rules in several datasets compiled by Shabalina et al., and the differences in siRNA efficacy assessment and quantification. Overall this analysis of how the LASSO model performs and looks like on independent datasets confirms that differences exist between datasets, but that a fair level of generality is reached both in terms of predictive accuracy and in terms of model interpretations with the model trained on Huesken's data.

## Discussion

**Figure 7:**
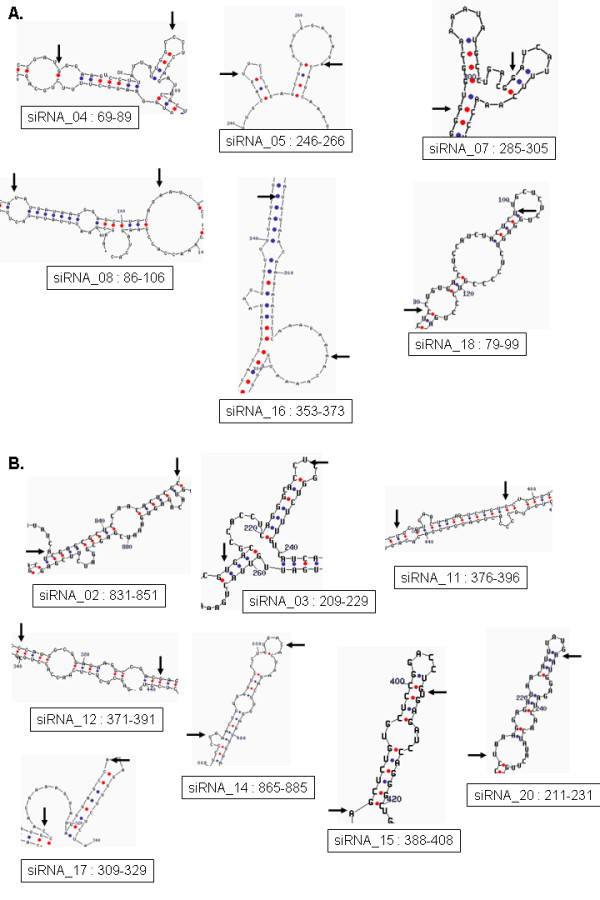
**Effect of target accessibility**. This picture shows the predicted structures of target sites sequences for the siRNAs that present the highest error between the experimental and the predicted value of activity. Local secondary structures were computed using the Mfold program. For each structure, an arrow indicates the begin position (right arrow) and the end position (left arrow) of the target site for the siRNA guide strand. Only local structures occurring with high abundance in the predictions issued by Mfold for a same target gene are shown (see Methods). A. Local target structures for siRNA sequences with an underpredicted activity. B. Local target structures for siRNA sequences with an overpredicted activity.

Despite considerable progress made recently siRNA design is still a subject of intense research. Ideally an efficient design should aim at defining a synthetic siRNA that mimics the natural RNA interference pathway considering different steps such as siRNA-RISC binding, duplex unwinding and strand selection, target specificity and accessibility, target cleavage and release. As each step likely involves multiple protein and nucleic acid interactions, key sequence and biophysical features are expected to be essential to ensure optimal functionality. In our study we focused only on intrinsic properties of siRNA, and it is likely that more precise prediction might be obtained by integrating information about the target specificity of the siRNA and the accessibility of the mRNA target, for example. As a first step towards assessing the importance of target accessibility, we considered the 20 siRNA sequences in our test set for which the discrepancies between predicted and actual efficacy were the largest (Table 7). In order to check whether there is any obvious structural basis for the errors of the model we computed the local secondary structure of the target mRNA with the Mfold program by submitting the corresponding fourteen target sequences provided by Huesken et al. [33] and subsequently focusing on the region of the mRNA containing the region targeted by the siRNA (Figure 7). Even though this dataset was produced using reporter constructs with fusion transcripts, a high correlation between potency profiles of siRNA against the reporter fusion mRNA and the corresponding endogenous gene was demonstrated suggesting that common sequence fragments in endogenous and fusion RNAs have similar secondary structures. For each predicted structure, the local free energy Δ*G*_*loc *_was calculated (see Methods) but conversely to Schubert et al. [20], no clear correlation between the actual silencing efficacy and the local energy of the target site structure was observed (Table 7). It is known that structure predictions have strong limitations when long RNAs are concerned, rendering only approximations of the actual folding at this site. Nevertheless, by a systematic inspection of the overall set of predicted local structures at the target site with a same range of Δ*G*_*loc*_, we observed that most target regions of siRNA with under-predicted efficacy have a 3' end in a stem loop (Figure 7A). This apparent target site accessibility could be related to the prominent role of nucleotides 2–8 on the 5' end of the siRNA guide strand that provide most of the binding energy that leads RISC to the target mRNA [39].

**Table 7:** List of siRNA with bad predictions.

Id	Target	Target site	M.A.	P.A.	status	Δ*G*_*loc*_	s.d	begin	end
1	NM_012864	CAGGCGCAGAAUUAUCUUAGG	1.341	0.765	under	-17	2.86	63	83
9		GUUUGCCGGAGACUGGAAAGC	0.019	0.373	over	-18.2	5.75	163	183
2	NM_017346	AUCGAGCGCUCCAACACUCGC	0.152	0.684	over	-19.9	0.00	831	851
3		UGACGCCACCUCAGGGCACCU	0.086	0.563	over	-17.7	0.00	209	229
11	NM_002559	UGCGUGAACUACAGCUCUGUG	0.420	0.743	over	-4.8	0.00	376	396
15		AGCUCUGUGCUCCGGACCUGU	0.419	0.713	over	-29.8	0.00	388	408
4	NM_003342	GGGAAGUCCUUAUUAUUGGCC	0.876	0.435	under	-17.9	0.06	69	89
5		UUCCUGAGCUGGAUGGAAAGA	1.201	0.799	under	-2.4	0.98	246	266
7	NM_016406	GUGGCAAAAUAUGCCUGACGG	0.999	0.616	under	-10.4	0.00	285	305
22		AUGCCUGACGGAUCAUUUCAA	1.173	0.907	under	-8.8	2.12	295	315
6	NM_003340	UUCUUUUAUCCAUUUGUUCAC	0.270	0.672	over	-7.2	1.41	255	275
8	NM_003347	UAUGAUAAGGGAGCCUUCAGA	1.099	0.728	under	-8.3	0.06	86	106
10	NM_018426 (XM_371822)	GAUGCCACCCGACGCCCUCAC	0.127	0.454	over	-9.5	1.18	2148	2168
12	NM_001009264 (XM_214061)	CCAGGGCGGAGAAGGCCGACG	0.239	0.548	over	-25.1	0.00	371	391
14		UGAACUUUGGGUCCCUGUGAC	0.268	0.568	over	-11.1	0.00	865	885
16	NM_001001481	UGUAACAAGAAUCCAAAGAAA	1.146	0.853	under	-10.9	0.19	353	373
17	NM_016021	CAACAAAAGGAGAGGGAGCCA	0.417	0.709	over	-19.7	0.00	309	329
18	NM_022005	CCUGUGACCUCCAUCUACUCU	0.968	0.682	under	-16.5	0.00	79	99
19	NM_007019	UGUAUGAUGUCAGGACCAUUC	0.321	0.601	over	-10.1	1.02	185	205
20	NM_006357	UAAAGGAGAUAACAUUUAUGA	0.520	0.795	over	-9.6	0.00	211	231

This table gives the list of siRNAs for which the discrepancy between the prediction and the actual potency were particularly large. Each siRNA sequence has been arbitrarily named from 1 to 22. Two sequences have been discarded because their target sequences have been modified in Genbank since Huesken's publication date. M.A. is the measured activity as given in Huesken et al. [33]. P.A. is the predicted activity according to our LASSO model. The column status refers to the activity of the siRNA that has been over- or under-predicted compared to the activity measured experimentally. The begin and end columns indicate the position of the siRNA guide strand within the target sequence. The Δ*G*_*loc *_corresponds to the mean local free energies of motifs in which nucleotides of the target sequence are involved for the 10 lowest energy structures and is given in kcal/mol. s.d. represents the standard deviation of each mean Δ*G*_*loc *_computed.

Conversely we observed a general trend for the mRNA targets of the siRNA with the most over-predicted efficacy to have a 3' extremity with a limited accessibility (Figure 7B). Although an effect due to the dataset used herein can not be completely excluded (see above), these preliminary observations suggest that a systematic and extended analysis of the local structures of target mRNA is required to decipher in more details the actual contribution of the local structural target accessibility to the silencing activity.

Regarding the importance of siRNA sequence features, the fact that the composite representation generally outperforms the sparse and spectral representation highlights a slight complementariness between them. Although the propensity of potent siRNAs to have particular nucleotides at particular positions has been recognized and biologically justified for some time, the importance of asymmetric short motifs along the sequence has been largely overlooked. The picture that emerges from these results is a superposition of different constraints to favor a good efficacy of siRNA, probably resulting from the necessity to follow different steps in the RNA interference pathway. The different constraints include in particular the preference for particular nucleotides at some positions, the presence of asymmetric motifs in the sequence, and a strong contrast between the free energies of the base pairs at the 5' and 3' ends of the double strand siRNA. Of particular interest is the important role played by the 3' overhang of the guide strand, first observed by Huesken et al. [33], who noticed an excess of G in position 21 in the 3' overhang of potent siRNAs. The analysis of the weights of the linear models based on the sparse-21 representation suggests that the main contribution of the overhang nucleotides is the negative effect of a C in position 21 of the guide strand, which has an average effect of decreasing the efficacy by 7% compared to a G in the same position, and by 6% compared to an A or a U. The pattern in position 20 is less important in quantitative terms, with a 3% improvement expected when a C is replaced by a U in this position. These findings suggest including 3' antisense nucleotide information in the siRNA design, and raises questions about the common inclusion of a TT 3' overhang in siRNA design. The role played by the 3' overhang nucleotides could be explained by recent findings about the human Argonaute 2 (Ago2) protein, a core constituent of the RISC complex characterized by two unique domains, PAZ and PIWI [36]. Structural studies of the PAZ domain suggest that it binds the two nucleotides at the 3' overhang of the siRNA duplex and is essential to guide the interaction between the siRNA and the target mRNA for cleavage and translational repression [40].

## Conclusion

In conclusion, we have developed an accurate and interpretable model for siRNA efficacy prediction that performs at least as well as the current state-of-art. We have shown how the weights estimated by the linear model provide a direct quantitative estimate of the effect of the features, and that most sequence features contribute either positively or negatively to the siRNA potency. Efforts towards publicly available siRNA datasets with confident annotation, albeit costly, is therefore highly desirable to improve current learning models. Finally, while considering steps involved in RNAi pathway, it clearly appears that other phenomena such as mRNA target accessibility and off-targets have to be considered into a single-one strategy for a fine tuned design and are currently under investigation.

## Methods

### Data representation

The main source of siRNAs used in this study is the dataset provided by Huesken et al. [33], comprising a total of 2431 randomly selected siRNAs targeting 34 different mRNAs and split into a training set of 2182 sequences and a test set of 249 sequences. Each siRNA sequence was converted to a vector of features using PYTHON scripts available from the authors.

### LASSO regression

Given a representation of siRNAs as vectors the LASSO procedure estimates a linear model with offset to explain a variable of interest (the siRNA efficacy in our case) from a set of siRNAs with known efficacy by minimizing the mean square error over the set of linear model with L1 norm bounded by a control parameter [34]. We used the LASSO implementation available in the Lars package [41] for the R statistical software. In each experiment the constraint on the L1 norm of the vector of weight was optimized by 5-fold cross-validation on the training set.

### Thermodynamic features

We considered two siRNA duplex thermodynamics indexes, a local stability and a base-stacking energy profiles [17], to check how features derived from prior biochemical knowledge perform compared to the sparse and spectral representation.

These profiles are based on free energy computation, a measure of the base pairing force. We used modified Freier tables [42-44] which provide an estimate of the base pair stack free energy Δ*G*_*Xi*,*Xi*+1 _for the nucleotide pair at positions *i *and *i *+ 1 of the sense (X = S) or antisense (X = A) strand, for *i *= 1, ..., 18. First, the local free energy profile is defined as the 18-dimensional vector whose *i*-th feature is precisely the free entropy Δ*G*_*Ai*,*Ai*+1_. Second, we consider the local stability profile, a 19-dimensional profile computed by taking sums of free energies over pentamers as described by the following equation for *i *= 1, ..., 19 [45] (Δ*G*_*penalty *_represents the penalty for *A *or *U *at 5' end, and ΔGXT
 MathType@MTEF@5@5@+=feaafiart1ev1aaatCvAUfKttLearuWrP9MDH5MBPbIqV92AaeXatLxBI9gBaebbnrfifHhDYfgasaacH8akY=wiFfYdH8Gipec8Eeeu0xXdbba9frFj0=OqFfea0dXdd9vqai=hGuQ8kuc9pgc9s8qqaq=dirpe0xb9q8qiLsFr0=vr0=vr0dc8meaabaqaciaacaGaaeqabaqabeGadaaakeaacqqHuoarcqWGhbWrdaWgaaWcbaGaemiwaG1aaSbaaWqaaiabdsfaubqabaaaleqaaaaa@31F7@ the free energy for the stack composed by the last position of X strand, and the dangling T):

LSi={∑j=1..4ΔGAj,Aj+1+ΔGAT+ΔGpenaltyfor i=1,∑j=i..i+3ΔGAj,Aj+1for i=i=2,...,15,∑j=20−i..20−i+3ΔGSj,Sj+1for i=16,...,18,∑j=1..4ΔGSj,Sj+1+ΔGST+ΔGpenaltyfor i=19,     (1)
 MathType@MTEF@5@5@+=feaafiart1ev1aaatCvAUfKttLearuWrP9MDH5MBPbIqV92AaeXatLxBI9gBaebbnrfifHhDYfgasaacH8akY=wiFfYdH8Gipec8Eeeu0xXdbba9frFj0=OqFfea0dXdd9vqai=hGuQ8kuc9pgc9s8qqaq=dirpe0xb9q8qiLsFr0=vr0=vr0dc8meaabaqaciaacaGaaeqabaqabeGadaaakeaaieqacqWFmbatcqWFtbWudaWgaaWcbaGae8xAaKgabeaakiabg2da9maaceqabaqbaeaabqGaaaaabaWaaabeaeaacqqHuoarcqWGhbWrdaWgaaWcbaGaemyqae0aaSbaaWqaaiabdQgaQbqabaWccqGGSaalcqWGbbqqdaWgaaadbaGaemOAaOMaey4kaSIaeGymaedabeaaaSqabaGccqGHRaWkcqqHuoarcqWGhbWrdaWgaaWcbaGaemyqae0aaSbaaWqaaiabdsfaubqabaaaleqaaaqaaiabdQgaQjabg2da9iabigdaXiabc6caUiabc6caUiabisda0aqab0GaeyyeIuoakiabgUcaRiabfs5aejabdEeahnaaBaaaleaacqWGWbaCcqWGLbqzcqWGUbGBcqWGHbqycqWGSbaBcqWG0baDcqWG5bqEaeqaaaGcbaGaeeOzayMaee4Ba8MaeeOCaiNaeeiiaaIaemyAaKMaeyypa0JaeGymaeJaeiilaWcabaWaaabeaeaacqqHuoarcqWGhbWrdaWgaaWcbaGaemyqae0aaSbaaWqaaiabdQgaQbqabaWccqGGSaalcqWGbbqqdaWgaaadbaGaemOAaOMaey4kaSIaeGymaedabeaaaSqabaaabaGaemOAaOMaeyypa0JaemyAaKMaeiOla4IaeiOla4IaemyAaKMaey4kaSIaeG4mamdabeqdcqGHris5aaGcbaGaeeOzayMaee4Ba8MaeeOCaiNaeeiiaaIaemyAaKMaeyypa0JaemyAaKMaeyypa0JaeGOmaiJaeiilaWIaeiOla4IaeiOla4IaeiOla4IaeiilaWIaeGymaeJaeGynauJaeiilaWcabaWaaabeaeaacqqHuoarcqWGhbWrdaWgaaWcbaGaem4uam1aaSbaaWqaaiabdQgaQbqabaWccqGGSaalcqWGtbWudaWgaaadbaGaemOAaOMaey4kaSIaeGymaedabeaaaSqabaaabaGaemOAaOMaeyypa0JaeGOmaiJaeGimaaJaeyOeI0IaemyAaKMaeiOla4IaeiOla4IaeGOmaiJaeGimaaJaeyOeI0IaemyAaKMaey4kaSIaeG4mamdabeqdcqGHris5aaGcbaGaeeOzayMaee4Ba8MaeeOCaiNaeeiiaaIaemyAaKMaeyypa0JaeGymaeJaeGOnayJaeiilaWIaeiOla4IaeiOla4IaeiOla4IaeiilaWIaeGymaeJaeGioaGJaeiilaWcabaWaaabeaeaacqqHuoarcqWGhbWrdaWgaaWcbaGaem4uam1aaSbaaWqaaiabdQgaQbqabaWccqGGSaalcqWGtbWudaWgaaadbaGaemOAaOMaey4kaSIaeGymaedabeaaaSqabaaabaGaemOAaOMaeyypa0JaeGymaeJaeiOla4IaeiOla4IaeGinaqdabeqdcqGHris5aOGaey4kaSIaeuiLdqKaem4raC0aaSbaaSqaaiabdofatnaaBaaameaacqWGubavaeqaaaWcbeaakiabgUcaRiabfs5aejabdEeahnaaBaaaleaacqWGWbaCcqWGLbqzcqWGUbGBcqWGHbqycqWGSbaBcqWG0baDcqWG5bqEaeqaaaGcbaGaeeOzayMaee4Ba8MaeeOCaiNaeeiiaaIaemyAaKMaeyypa0JaeGymaeJaeGyoaKJaeiilaWcaaaGaay5EaaGaaCzcaiaaxMaadaqadaqaaiabigdaXaGaayjkaiaawMcaaaaa@E875@

### RNA secondary structure prediction

To assess mRNA target accessibility, we computed local RNA secondary structures using the program mfold version 3.1 [43,46] with the following parameters W = 0, MAXBP = 100, P = 100 and MAX = 10 [47]. For each target sequence, the ten first predicted structures with the lowest free energies were recorded. For each predicted, local free energies Δ*G*_*loc *_were calculated for the structural motives in which nucleotides of the target site were involved. Each local target sequence was carefully inspected and those that occurred at high abundance (from 7 to 10/10) with associated Δ*G*_*loc *_values in the same range (+/- 0.2 kcal/mol) was selected and subsequently considered.

## Availability and requirements

The algorithm is implemented in the platform-independent web server DSIR (Design of SIRna), freely available without restriction for academic and non-academic users at .

## Authors' contributions

All authors conceived the experiments. JPV and NF implemented the algorithms and performed the experiments. YV and NF studied the structures of siRNA. JPV and YV drafted the manuscript. All authors read and approved the final manuscript.
